# The Rate Stabilizing Tool: Generating Stable Local-Level Measures of Chronic Disease

**DOI:** 10.5888/pcd16.180442

**Published:** 2019-03-28

**Authors:** Harrison Quick, Joshua Tootoo, Ruiyang Li, Adam S. Vaughan, Linda Schieb, Michele Casper, Marie Lynn Miranda

**Affiliations:** 1 Department of Epidemiology and Biostatistics, Drexel University, Philadelphia, Pennsylvania; 2 Children’s Environmental Health Initiative, Rice University, Houston, Texas; 3 Division for Heart Disease and Stroke Prevention, Centers for Disease Control and Prevention, Atlanta, Georgia

## Abstract

Accurate and precise estimates of local-level epidemiologic measures are critical to informing policy and program decisions, but they often require advanced statistical knowledge, programming/coding skills, and extensive computing power. In response, we developed the Rate Stabilizing Tool (RST), an ArcGIS-based tool that enables users to input their own record-level data to generate more reliable age-standardized measures of chronic disease (eg, prevalence rates, mortality rates) or other population health outcomes at the county or census tract levels. The RST uses 2 forms of empirical Bayesian modeling (nonspatial and spatial) to estimate age-standardized rates and 95% credible intervals for user-specified geographic units. The RST also provides indicators of the reliability of point estimates. In addition to reviewing the RST’s statistical techniques, we present results from a simulation study that illustrates the key benefit of smoothing. We demonstrate the dramatic reduction in root mean-squared error (rMSE), indicating a better compromise between accuracy and stability for both smoothing approaches relative to the unsmoothed estimates. Finally, we provide an example of the RST’s use. This example uses heart disease mortality data for North Carolina census tracts to map the RST output, including reliability of estimates, and demonstrates a subsequent statistical test.

**Summary**

**What is already known on this topic?**

Existing methods of generating small area estimates often require advanced statistical knowledge, programming and coding skills, and extensive computing power.

**What is added by this report?**

We created an ArcGIS Tool — the Rate Stabilizing Tool (RST) — that produces age-adjusted rate estimates from record-level data and indicates which rates should be considered statistically reliable. This tool is particularly important for generating estimates when the population size or the number of events is small. With the RST, estimates can be generated for a wide range of geographic units, including subcounty levels.

**What are the implications for public health practice?**

With its ease of use, the RST addresses the need to produce stable local estimates of chronic disease measures to improve chronic disease surveillance, prevention, and treatment.

## Introduction

Public health professionals are increasingly using spatial analysis and geographic information systems (GIS) to document and address geographic disparities in the burden of chronic disease (1–8). Maps of local-level disparities in chronic disease morbidity, mortality, risk factors, and treatments are critical to informing policy and program decisions and enhancing partnerships to address the disparities (9–12). One important component in the use of GIS for chronic disease prevention and health promotion is the availability of data at the local level (eg, county, census tract) that yield stable estimates that are both accurate and precise. Here, our focus is the ability to produce stable *event* rates (eg, death rates), which depend primarily on the number of events that occur in a place of interest for a designated period. These event counts in turn depend on the prevalence or incidence of the event and the population size. In general, the smaller the population size, the smaller the event counts and the greater the instability in population measures of chronic disease. In particular, small counts are often encountered when analyzing small geographic areas (eg, census tracts) or examining population subgroups (eg, race/ethnicity, sex) or sparsely populated regions (eg, rural areas). In this article, we use the term “small area” to refer to areas for which the data alone do not provide stable estimates for a given population measure, regardless of the physical size of the geographic area itself.

Recent advances in computing and in the field of small-area estimation — specifically Bayesian methods (13–17) — have provided avenues for generating more reliable local-level population measures of chronic disease when the number of events are small. In particular, these approaches often involve *smoothing* observed rates toward a common mean (eg, the national average) or toward neighboring values. However, these methods typically require knowledge of advanced statistics, programming/coding skills, and extensive computing power — resources that may be challenging to obtain for many public health professionals in need of stable small-area estimates.

In response to the need for local-level measures of chronic disease and recognizing the challenges that often exist in generating reliable estimates, we developed the Rate Stabilizing Tool (RST). The RST is an ArcGIS-based tool that enables users to input their own record-level data to generate more reliable age-standardized measures of chronic disease (eg, prevalence, rates) or other population health outcomes at the local level. Bayesian modeling techniques are built into the tool, enabling users to better evaluate measures of statistical uncertainty for each population subgroup and locale.

In this article, we describe the statistical techniques that are built into the Rate Stabilizing Tool, review the results from a simulation study, provide an overview for how to use the RST, and discuss its strengths and limitations. Files needed to install the RST and detailed instructions are available at https://www.cdc.gov/dhdsp/maps/gisx/rst.html. Statistical and technical details of the Rate Stabilizing Tool are available in a Web Appendix (https://sites.google.com/site/harryq/rst).

## Statistical Techniques of the Rate Stabilizing Tool

### Bayesian modeling

The Rate Stabilizing Tool employs Bayesian modeling techniques to generate local-level estimates of the prevalence of chronic disease (or other outcomes). These estimates are more stable than those generated by conventional methods. Bayesian modeling techniques are used because 1) they are well-equipped to maximize the information gained from available data in situations where data are sparse, thereby yielding estimates with greater precision than crude estimates, and 2) they generate accompanying measures of uncertainty, the benefits of which will be discussed shortly. Bayesian methods generate estimates by combining information from the observed data (via the *likelihood* [ie, the distribution of the observed data given various model parameters]) and so-called *prior information* (often expressed in the form of model structure [eg, spatial correlation]). The result of this combination is referred to as the *posterior distribution*. From the posterior distribution, we can then generate summaries such as the mean and 95% credible interval (the Bayesian equivalent of classical confidence intervals) for each of the region-specific rate estimates and make statistical comparisons with other values. An extended introduction to Bayesian methods is available in the Web Appendix; a more thorough introduction to Bayesian methods can be found in the text by Carlin and Louis (18).

Two forms of Bayesian modeling are incorporated into the RST — a nonspatial approach and a spatial approach. In the nonspatial approach, local-level rates are smoothed toward the observed rate from the overarching spatial domain (eg, the rate for a selected state). In contrast, the spatial approach smooths each local-level rate toward the crude rate of the combined neighboring geographic units (and is similar to the approach of Clayton and Kaldor [17]). Complete details on these approaches, including justifications for the selected likelihood and prior distributions and derivations of the posterior distributions, are available in the Web Appendix.

### Age-standardization of local-level rates

Age-standardization of local-level chronic disease rates is important because differences in age-distributions across regions can contribute to stark differences in measures of the burden of chronic disease, even if the underlying rates in each age-group are comparable. Generally speaking, age-standardized rates for a given region are obtained by computing the weighted average of the region’s age-specific rates, where the weights used are based on the age distribution of a standard population (eg, the 2010 US standard [19,20]). Directly using these age-specific rates poses challenges, however, because crude estimates of these rates are often based on small counts. Not only can these small counts lead to age-specific rate estimates that are unstable, but the instability in the age-specific rates can seep into the age-standardized estimates. As such, a key feature of the RST is that we first obtain smoothed estimates of the age-specific rates by using one of the aforementioned Bayesian methods, and then these smoothed age-specific rates are used to compute the age-standardized rates for each region. This process allows the uncertainty in the smoothed age-specific rates to propagate through to the age-standardized rates; in contrast, estimates of the age-standardized rates based solely on the data may require complex equations to approximate these variance estimates (21,22).

## Simulation Study

We conducted a simulation study to compare smoothed age-standardized rates (both spatial and nonspatial smoothing) with unsmoothed age-standardized rates to demonstrate the RST’s effectiveness. The simulation study was based on heart disease death data from US counties for 1979–1988 and multiple age groups (35–44, 45–54, 55–64, 65–74, 75–84, and ≥85) obtained from CDC WONDER (23). From these data, we calculated an estimate of the age-group–specific mortality rate for each county; these are henceforth considered the “true rates” and were used to generate 100 data sets of simulated death count. We then analyzed the simulated death data by using the spatial and nonspatial smoothing methods of the RST and compared the estimates from the RST to the unsmoothed age-standardized mortality rates. We compared estimates from all 3 approaches by using root mean square error (rMSE), a measure that combines the bias of an estimate and its variance, and we estimated coverage probabilities (ie, the proportion of the 95% credible interval that contains the true rates) for both smoothing approaches. Complete details of the simulation study are available in the Web Appendix.

**Figure 1:**
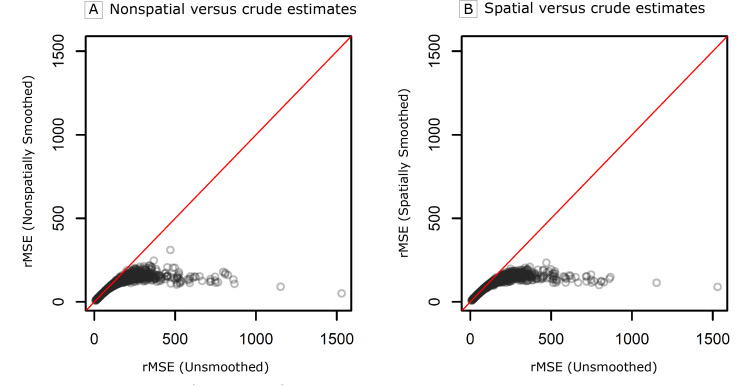
Comparison of the root mean square error (rMSE) of the age-standardized rates from the 2 smoothing approaches (A, nonspatial vs crude estimates and B, spatial vs crude estimates) of the Rate Stabilizing Tool to the unsmoothed rates estimated directly from the raw data in the simulation study.

Figure 1 compares the rMSE of the age-standardized rate estimates from the spatial and nonspatial smoothing approaches with the rMSE of the unsmoothed rates, where a lower rMSE indicates better compromise between accuracy (ie, bias) and stability (ie, variance). Here, we see the key benefit of smoothing, namely a dramatic reduction in the rMSE for both smoothing approaches when compared with the unsmoothed estimates. A comparison of the age-standardized and age-group specific estimates from the 2 smoothing approaches shows only minor differences. A more thorough comparison of these 2 approaches, including maps of the rMSEs of the age-group specific and the age-standardized rates, can be found in the Web Appendix. In addition to improvements in rMSE, both smoothing approaches achieved coverage probabilities approximately equal to 0.95 as desired (ie, the 95% credible intervals contain the true values approximately 95% of the time).

## An Overview of How to Use the Rate Stabilizing Tool (RST)

The RST operates as a set of tools within an ArcToolbox toolset; no installation or administrative privileges are required to run this tool. After inputting individual-level data into ArcGIS, users specify their desired age structure, and then the RST produces 3 sets of age-standardized rates: unsmoothed; nonspatially smoothed; and spatially smoothed. The RST also generates 95% credible intervals and alerts on the reliability of each smoothed rate estimate. An overview of the use of the tool is as follows:

**1. Input individual-level data**. The user loads a table where each record represents a single event (eg, death) and contains the individual’s age and a geographic identifier (eg, census tract, county).

**2. Choose age structure**. The user then selects age groups that will be used for age-standardization. For age standardization, the RST connects to the US Census Data web API (https://census.gov/data/developers/data-sets/acs-5year.html) and downloads the age-specific population sizes for each census geography of interest, along with the age distribution for the US standard population.

**3. Import US Census areal unit boundary definitions (** 24 **) (eg, a shapefile) for map creation and spatial smoothing**. In addition to facilitating the creation of maps, the tool will use the boundary definitions to create a neighborhood dictionary for the geographic units in the spatial domain. The neighborhood dictionary is required for RST’s spatial smoothing approach. This dictionary describes which geographic units are adjacent to one another, thus defining the neighbor pairs. Once constructed, the neighborhood dictionary is saved and can be re-used for future analyses with the same shapefile.

**4. Examine and evaluate the output.** The RST generates an output text file, with one record for each geographic unit. Each record contains the following information:

- Age-standardized rate, unsmoothed
- Age-standardized rate, smoothed (nonspatial) and corresponding 95% credible intervals
- Age-standardized rate, smoothed (spatial) and corresponding 95% credible intervals

In addition to providing rate estimates and 95% CIs, the RST also provides an alert when the estimate for a given geographic unit is deemed unreliable (ie, when the width of the 95% credible interval is larger than the estimate). The RST generates 3 types of alerts:

- Unreliable nonspatial Bayesian estimate, when the nonspatial Bayesian estimate is not reliable for a given geographic unit;
- Unreliable spatial Bayesian estimate, when the spatial Bayesian estimate is not reliable for a given geographic unit; and
- Unreliable estimate, when neither of the Bayesian estimates are reliable for a given geographic unit.

**5. Mapping the results.** After evaluating the output from the RST and deciding which values are appropriate to display on a map, users can create maps by joining the output from the RST to their US Census areal unit boundary definition shapefile for the area of interest. Users can easily make maps comparing the display of the 3 types of rates generated by the RST.

**6. Using the tool: an example.** To illustrate the use of the RST, we analyzed data on heart disease deaths in Charlotte, North Carolina, for 2006–2011. We used the RST to age standardize the mortality rates to the 4 age groups (0–34, 35–44, 45–64, and ≥65 y) and generated heart disease mortality rates at the census tract level. We obtained shapefiles corresponding to these boundaries from 2010 US Census Topologically Integrated Geographic Encoding and Referencing reference files. These boundaries were used in the RST’s spatial Bayesian smoothing approach.

**Figure 2:**
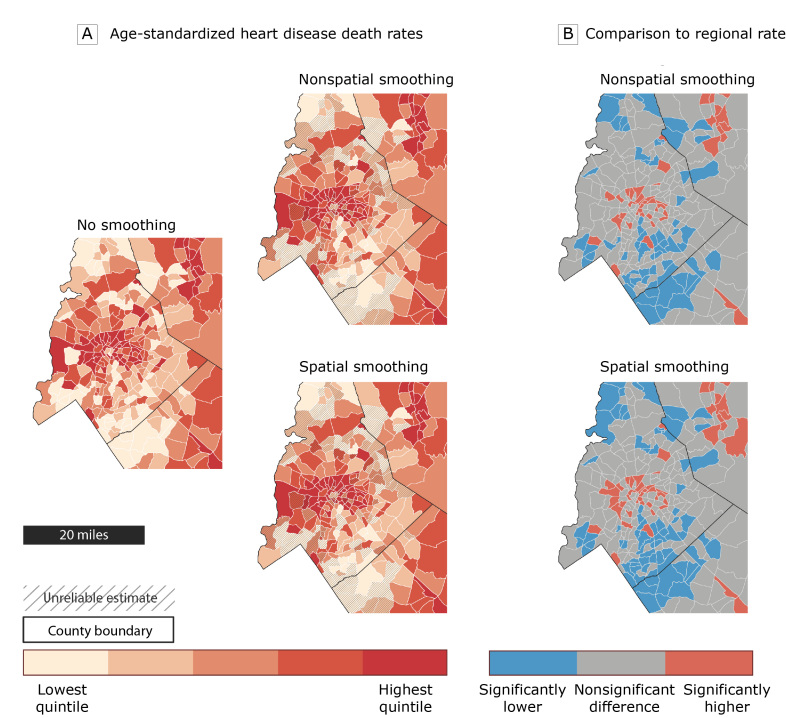
Illustration of the functionality of the Rate Stabilizing Tool using heart disease mortality data from the region surrounding Charlotte, North Carolina. A, Age-standardized heart disease death rates by census tract using 3 methods, with hatch marks indicating unreliable rates based on the 2 Bayesian smoothing approaches. B, census tracts with death rates that are significantly higher or lower than the state rate using the 2 smoothing methods.

The map on the left side of Figure 2A displays unsmoothed age-standardized heart disease mortality rates in Charlotte and the surrounding area. Although this map highlights census tracts with high and low observed mortality rates, it obscures the degree of statistical uncertainty in these rates. For example, if a priority is to target public health interventions to areas with elevated rates, how would one differentiate between census tracts with truly high rates and census tracts with high rates that are unreliable because of small population sizes? To address this challenge, we mapped the smoothed rates (nonspatially smoothed and spatially smoothed) and found census tracts with unreliable mortality rates (2 maps on right side of Figure 2A). These 2 maps indicate that the rates for many of the census tracts are unreliable (33.4% with nonspatial smoothing and 34.1% with spatial smoothing) and should be considered with caution.

An additional way to use the information on statistical uncertainty generated by the RST is to compare the rate for each census tract to a regional standard. The maps in Figure 2B display census tracts that have age-standardized heart disease death rates that are significantly higher or significantly lower than the regional average rate based on the 95% credible intervals generated by the RST for spatially and nonspatially smoothed rates. Census tracts where the 95% credible intervals do not include the mean rate for the region were classified as having rates that were significantly higher or significantly lower than the rate for the region. For several census tracts — such as those in the southern part of the Charlotte, North Carolina region (right side of Figure 2A) — the rates were determined to be unreliable because of the wide 95% credible intervals, but we can conclude that those rates are significantly below the regional average because the entire range of the 95% credible intervals is below the regional rate.

## Strengths and Limitations

An important strength of the RST is that it combines 2 tasks — rate smoothing and age-standardization — into a single tool. By doing so, the RST avoids the potential pitfall of estimating age-standardized rates from extreme age-specific rates (eg, rates based on zero deaths). The RST overcomes this pitfall by first smoothing the age-specific rates, producing age-specific rates that are more reliable than those calculated directly from the data. By calculating the age-standardized rates on the basis of these smoothed rates, we can improve the stability of our estimates (Figure 1).

In addition to the ease-of-use attributable to combining these 2 tasks, the RST offers inferential improvements. We demonstrated through our simulation study that both approaches for computing smoothed age-standardized rates dramatically improve the quality of the estimates compared with the estimates generated solely from the observed data based on the rMSE. In addition, the RST provides 95% credible intervals for smoothed age-standardized rates: this is a notable strength given the complexity of producing uncertainty estimates when calculating age-standardized rates according to standard methods (21,22). Furthermore, the 95% credible intervals produced by the RST yield coverage probabilities (ie, the probability that the 95% credible interval contains the true value) near the desired 0.95 for both the age-specific and the age-standardized rate estimates. This indicates that convenience of the RST does not compromise statistical validity.

This version of the RST has several limitations. First, although many public-use data sets consist of aggregate, tabular data that comprise the number of events and the population sizes stratified by geographic unit and age group, the RST is designed only to analyze *record-level* data. To mitigate this limitation, we developed instructions to generate *synthetic* individual-level data from a table of aggregate data. Future iterations of the tool will allow users to import record-level or tabular data directly. In addition to added flexibility, future updates to the tool will facilitate the analysis of public-use data sets from sources such as CDC WONDER, which are subject to various privacy protections that result in data *suppression* (eg, CDC WONDER suppresses counts of ≤9 to protect data privacy [25]); ignoring (or inappropriately accounting for) these protective measures may result in biased rate estimates (26). After this functionality is added, the RST will be able to seamlessly account for such privacy protections to produce rate estimates for small areas that are both reliable and valid; Quick et al (27) explained how this can be done. The RST is also not currently equipped to analyze survey data, where accommodating sample sizes and survey weights adds layers of complexity that must be carefully considered.

A final limitation of the RST is that it relies on empirical Bayesian methods rather than fully Bayesian methods. The approaches used by the RST smooth toward estimates determined by the data and the degree of smoothing is predetermined. In contrast, a fully Bayesian approach would include prior distributions on the values each region is smoothed toward *and* the degree of smoothing, thereby learning from the data what each region should be smoothed toward and how strong the smoothing should be. The conditional autoregressive model of Besag et al (13) is a popular approach for this type of analysis. Unfortunately, fully Bayesian methods have one key drawback: computational burden. In particular, fully Bayesian models are typically fitted by using complex Markov chain Monto Carlo algorithms that must be run until convergence has been achieved. That is, the algorithm needs to iteratively learn about each of the model parameters until their estimates stabilize, a process which often requires thousands of iterations and can take minutes or hours to complete depending on the size of the data set. Because convergence is often diagnosed visually, designing the RST to diagnose convergence in an automated and efficient fashion is much more challenging. Despite these computational challenges, however, the inferential benefits of fully Bayesian models necessitate their consideration in future iterations of the RST.

## Conclusion

The Rate Stabilizing Tool is an add-on tool for ArcGIS that produces accurate and precise estimates of event rates for geographic areas with small population sizes or small counts. The RST imports record-level event data and uses an empirical Bayesian model to estimate age-standardized rates and 95% credible intervals for user-specified geographic units. In addition, users are alerted if a point estimate is deemed unreliable for a given geographic unit. With its ease of use, the RST addresses the need to produce stable local estimates of chronic disease measures to improve chronic disease surveillance, prevention, and treatment.
